# Accuracy of Micro-Computed Tomography in Post-mortem Evaluation of Fetal Congenital Heart Disease. Comparison Between Post-mortem Micro-CT and Conventional Autopsy.

**DOI:** 10.3389/fped.2019.00092

**Published:** 2019-03-22

**Authors:** Camilla Sandrini, Lucia Rossetti, Vanessa Zambelli, Roberta Zanarotti, Franca Bettinazzi, Roberta Soldá, Concetta Di Pace, Stiljan Hoxha, Flavio Luciano Ribichini, Giuseppe Faggian, Claudio Lombardi, Giovanni Battista Luciani

**Affiliations:** ^1^Division of Cardiology, Department of Medicine, University of Verona, Verona, Italy; ^2^School of Medicine and Surgery, University of Milano Bicocca, Monza, Italy; ^3^Azienda Ulss 9 Scaligera, Verona, Italy; ^4^Division of Obstetrics and Gynecology, Department of Surgery, Dentistry, Pediatrics and Gynecology, University of Verona, Verona, Italy; ^5^Department of Pathology, Department of Diagnostics and Public Health, University of Verona, Verona, Italy; ^6^Division of Cardiac Surgery, Department of Surgery, Dentistry, Pediatrics and Gynecology, University of Verona, Verona, Italy; ^7^Studio Diagnostico Eco srl, Vimercate, Italy

**Keywords:** congenital heart disease—cardiac, post mortem micro-computed tomography, prenatal diagnosis, fetal echocardiography, early prenatal diagnosis

## Abstract

**Aims:** Early prenatal diagnosis of congenital heart disease is feasible. Conventional autopsy is the current gold standard method for post-mortem confirmation. Radiologic techniques alternative to conventional autopsy, such as post-mortem micro-computed tomography, have been proposed in case of limited diagnostic accuracy (i.e., early termination of pregnancy, samples of small dimension or of low weight). The aim of the present study was to define accuracy of micro-computed tomography for post-mortem diagnosis of congenital heart disease in gross anatomy samples.

**Methods and Results:** Fetal heart underwent *in-utero* prenatal echocardiography and *ex-vivo* post-mortem evaluation by 9 μm resolution micro-computed tomography and conventional autopsy. For each case, 25 indices of cardiac anatomy were studied by post-mortem micro-computed tomography and conventional autopsy; these were used to compare the two post mortem techniques. Ten samples were examined (gestational age between 12 + 4 and 21 + 6 weeks of gestation). Considering comparable indices, agreement between post-mortem micro-computed tomography and conventional autopsy was of 100% and sensitivity and specificity were of 100%. In “challenging specimens,” post-mortem micro-computed tomography diagnoses more indices as compared to conventional autopsy and 84% of “not-diagnostic” indices at conventional autopsy would be diagnostic at post-mortem micro-computed tomography.

**Conclusion:** Micro-computed tomography can be a valid diagnostic alternative to conventional autopsy for post-mortem evaluation of human fetal heart. In addition, it may prove superior to conventional autopsy particularly in cases coming from early termination of pregnancy or in samples of small dimension or of low weight.

## Introduction

Congenital heart disease is the most frequent congenital malformations. Early prenatal diagnosis of congenital heart disease is feasible, thanks to progress in technology, and to better understanding of risk factors for heart malformations (1–6). By combining 2D analysis and color Doppler techniques, the four chambers, the outflow tracts and the aortic and ductal arches can be seen in a percentage of cases as early as 8–9th week while a complete, or near complete, fetal cardiac assessment can be reached at 11th week (1). Early diagnosis of congenital heart disease is important because it promotes the research of extra-cardiac pathology, it allows physicians to study the possible progression of the disease in order to potentially offer earlier intervention and it enables families to have multidisciplinary counseling. In case of fetal demise or parental decision for termination of pregnancy, post-mortem examination of the fetal heart is important for familial counseling, educational and research purposes. Conventional autopsy, the current gold standard technique for *ex vivo* confirmation of congenital heart disease, is an invasive and destructive tool and it can be challenging, up to impracticable, even for expert pathologists, especially in case of specimens low for gestational age, dimension, or weight (7–9). To overcome possible gaps in post-mortem evaluation of fetal heart due to limitations of conventional autopsy, different radiologic techniques have been proposed [post-mortem contrast-enhanced micro computed tomography (micro-CT) (10–13), post-mortem cardiac magnetic resonance imaging (10, 14–16), post-mortem CT-angiography (10), post-mortem X-ray phase-contrast tomography (10)]. Post-mortem micro-CT of the fetal heart has been tested in *ex vivo* murine and human cardiac samples, showing feasibility and high concordance rate with conventional autopsy. Given its high resolution power (up to 9 μm), it is feasible even at very early gestational age (7th week) and in samples of small dimension or of low weight, where autopsy can miss diagnosis and cardiac details (11–13). Though currently not validated, they may become valid alternative to autopsy for *ex vivo* cardiac examination, even in case where autopsy lacks of diagnostic power.

This study compares post-mortem micro-CT and conventional autopsy of the human fetal heart in case of prenatal suspected congenital heart disease. The primary aim of the study is to define whether micro-CT is at least equal to conventional autopsy in post-mortem diagnosis of congenital heart disease. The secondary aim of the study is to define if micro-CT is more accurate as compared to conventional autopsy in analyzing samples coming from early termination of pregnancy (<16 weeks of gestation) or in samples of small dimension (1 cm or less) or low weight (1 g or less). In this subgroup of patients, we hypothesize that micro-CT has greater diagnostic power when compared to conventional autopsy.

## Methods

### Selection and Preparation of Specimens

Cases were selected prospectively. Inclusion criteria for study were fetal echocardiography performed at our Center, prenatal diagnosis of congenital heart disease, decision for termination of pregnancy, agreement to participate to study. For each case, there was fully informed parental consent for use of samples for research. For each case, anamnestic data on pregnancy, fetal extra-cardiac malformations and genetics were collected and anonymized. Especially in case of suction aspiration (termination of pregnancy before the 14th week of gestation), fetal body could be too damaged to permit whole body fetal micro-CT and autopsy. Therefore, in order to be able to compare specimens coming from all gestational ages, samples consisted only in isolated fetal hearts (or heart-lungs in case of prenatal suspicion of heterotaxy syndrome). A single pathologist with expertise in the field of fetal-placental disease isolated the samples. Post-mortem evaluation consisted in micro-CT followed by conventional autopsy. Between termination of pregnancy and micro-CT and between micro-CT and conventional autopsy, all specimens should be left in solution with formalin 10% to preserve anatomic characteristics and tissue properties. Post-mortem micro-CT can easily image calcified tissue but cannot differentiate between soft tissues because of their similarity in x-ray attenuation. As soft tissues adsorb different concentrations of iodine solution, contrast agent enables micro-CT to differentiate between soft structures. Therefore, in order to prepare samples for post-mortem micro-CT, they were removed from formalin 10%, weighted, immersed in Lugol solution at 20, 25, or 50% for 72 h and then washed to remove excess surface liquid just before micro-CT scan, according to previously described protocols (12). After micro-CT scan, they were re-weighted to estimate shrinking effect on tissue and then re-immersed in formalin 10%.

### Techniques

Prenatal *in vivo* fetal echocardiographies were performed by a single pediatric cardiologist with expertise in the field of fetal cardiology using a Voluson E8 with a 2–7 MHz convex probe (General Elettrics Healthcare). Each exam consisted in all requested scans according to Italian guideline (2). Segmental approach (17) was used to study the heart.

Post-mortem micro-CTs were performed using a micro-CT scanner SkyScan 1176 (Bruker, Kontich, Belgium) by a single radiologist with expertise in the field of human heart analysis. Smaller voxel reached resolution of 9 μm. Acquisition dataset changed for each specimen, according to sample properties. Post-processing analysis was performed using CTVox volume rendering 64 bit version, DATAVIEWER 64 bit version (Bruker, Kontich, Belgium) and Horos v2.4 software (free and open source code software at Horosproject.org). Segmental approach (17) was used to study the heart.

Conventional autopsies were performed by a single senior pediatric cardiac surgeon using 3.5 magnification loops and neonatal surgical instruments. A standard technique to dissect the heart was adopted (inflows, atrioventricular valves, ventricles, semilunar valves, great arteries, aortic arch, and branches). Segmental approach (17) was used to study the heart.

### Data Analysis

For each case, twenty-five indices of cardiac anatomy derived from conventional segmental analysis of the heart (17) were studied and were used to compare post mortem micro-CT and conventional autopsy (atrial situs, ventricular loop, relationship of the great arteries, atrio-ventricular connection, ventricular-arterial connection, systemic venous returns, pulmonary venous returns, atrial septum, right atrium, left atrium, tricuspid valve, mitral valve, ventricular septum, right ventricle, left ventricle, right ventricular outflow tract, left ventricular outflow tract, pulmonary valve, main pulmonary artery, right pulmonary artery, left pulmonary artery, aortic valve, aortic root, aortic arch, arterial duct). For each index, a technique is defined “diagnostic” when it is able to define that index as normal or abnormal and “not-diagnostic” when it is inconclusive and it can neither confirm nor deny the presence of that index. Pulmonary venous returns was defined normal if at least 2 pulmonary veins were seen terminating in left atrium. Conventional autopsy was used as gold standard technique. For indices that are evaluable by both techniques, we defined “concordance” when the two compared techniques gave the same result for the studied index. Therefore, we included in “concordance” the true positive and the true negative indices. On the other hand, we defined “discordance” when the two compared techniques gave different result for the studied index. Therefore, we included in “discordance” the false positive and the false negative indices. For “not-diagnostic” indices, we define “missense of micro-CT” if that index was deemed “not-diagnostic” with micro-CT and “diagnostic” with conventional autopsy and “apparent advantage of micro-CT” if that index was deemed “diagnostic” with micro-CT but “not-diagnostic” with conventional autopsy.

We arbitrarily defined “challenging specimens” samples coming from early termination of pregnancy (gestational age ≤16th week), samples of small dimension (1 cm or less) or low weight (1 g or less).

As little is known about comparison between post-mortem micro-CT and conventional autopsy in *ex vivo* human fetal heart affected by congenital heart disease coming from early and late termination of pregnancy, we decided not to blind prenatal data to radiologist and pathologist and results of micro-CT to pathologist.

Statistical analysis relates only to concordant and discordant indices. Data are given as agreement, sensitivity and specificity. “Not-diagnostic” indices are only described in the text. “Diagnostic power” refers to the ability of a technique in defining indices. We define “greater diagnostic power” of micro-CT when it is able to define more indices as compared to conventional autopsy.

## Results

Ten cases met the eligibility criteria. Technical data on post-mortem micro-CT are summarized in Table 1. Data on prenatal information and macroscopic dissection are summarized in Table 2. Median gestational age at prenatal diagnosis was 16 weeks (17 ± 3 weeks, range 13–21 weeks). Median gestational age at termination of pregnancy was 18 weeks (17 ± 3 weeks, range 14–22 weeks). Mean transverse and longitudinal diameters were respectively 1.02 ± 0.42 cm (range 0.5–1.8 cm) and 1.18 ± 0.41 cm (range 0.5–1.7 cm). Mean weight before and after micro-CT were respectively 1.88 ± 1.43 g (range 0.39–4 g) and 1.35 ± 1.11 g (range 0.12–3.21 g). Mean time interval between termination of pregnancy and post-mortem micro-CT was 80 ± 94 days (range 16–260 days). Mean time interval between termination of pregnancy and conventional autopsy was 127 ± 99 days (range 51–313 days). Mean time interval between post-mortem micro-CT and conventional autopsy was 47 ± 7 days (range 35–53 days).

**Table 1:** Post-mortem micro-CT properties for images acquisition.

**Case**	**% Lugol**	**Hours in lugol**	**Filter**	**Resolution (μm)**	**Exposure time (msec)**	**Energy range (V)**	**Current range (μA)**	**Rotational step (^**°**^)**
1	25	72	Cu+Al	18	500	89	264	0.50
2	25	72	Cu+Al	18	500	89	264	0.50
3	25	72	Cu+Al	18	500	89	264	0.50
4	20	72	Cu+Al	18	500	89	264	0.50
5	25	72	Cu+Al	18	500	89	264	0.50
6	20	72	Al 0.5 mm	18	210	50	500	0.50
7	20	72	Al 0.5 mm	9	900	50	500	0.30
8	20	72	Al 0.5 mm	18	210	50	500	0.50
9	25	72	Cu+Al	18	500	89	264	0.50
10	20	72	Al 0.5 mm	9	900	50	500	0.30

**Table 2:** Data on macroscopic dissection.

**Case**	**GA diagnosis (w+d)**	**GA TOP (w+d)**	**Longitudinal diameter (cm)**	**Transverse diameter (cm)**	**Weight pre micro-CT (g)**	**Weight post micro-CT (g)**
1	20	20+4	1.50	1.30	3.37	2.42
2	16+2	18+6	1.50	1.30	3.07	2.21
3	20+2	20+4	1.50	1.20	3.33	2.18
4	16+1	16+5	1.00	0.80	1.32	0.83
5	21+2	21+6	1.70	1.80	4.00	3.21
6	16+2	16+3	1.10	0.80	0.65	0.45
7	12+4	13+6	0.60	0.50	0.39	0.18
8	14+3	14+3	1.00	0.70	0.49	0.37
9	17+2	18+2	1.40	1.30	1.80	1.55
10	13+2	13+4	0.50	0.50	0.40	0.12

*GA, gestational age; w, weeks; d, days; TOP, termination of pregnancy; cm, centimeter; g, gram*.

Table 3 summarizes distribution of indices and statistical data.

**Table 3:** Statistical analysis.

	**Conventional autopsy**		
**Micro-CT**	**Positive (abnormal)**	**Negative (normal)**	**“Not-diagnostic”**
Positive (abnormal)	24	0	3
Negative (normal)	0	150	55
“Not-diagnostic”	4	1	13
	Sensitivity	Specificity	Agreement
	100%	100%	100%

Overall, 174/250 (69.6%) indices were evaluable by both techniques. They comprise concordant (174/174, 100%) and discordant (0/174, 0%) indices. The agreement between micro-CT and conventional autopsy was 100%. There were no false positive and false negative indices in micro-CT as compared to conventional autopsy (sensitivity and specificity of post-mortem micro-CT 100%).

76/250 (30.4%) indices were deemed “not-diagnostic” at micro-CT, conventional autopsy or both techniques. They comprise 5 “missense of micro-CT” indices (4 indices “not-diagnostic” at micro-CT and abnormal at conventional autopsy and 1 index “not-diagnostic” at micro-CT and normal at conventional autopsy), 58 “apparent advantage of micro-CT” indices (3 indices abnormal at micro-CT and “not-diagnostic” at conventional autopsy and 55 indices normal at micro-CT and “not-diagnostic” at conventional autopsy) and 13 indices “not-diagnostic” at both micro-CT and conventional autopsy (Table 4).

**Table 4:** Diagnoses and missing data.

**Case**	**ND micro-CT /abnormal autopsy**	**ND micro-CT /normal autopsy**	**Abnormal micro-CT /ND autopsy**	**Normal micro-CT /ND autopsy**	**ND micro-CT /ND autopsy**
1				SVR, PVR	AD, VS
2				SVR, PVR	AD
3	RVOT, LVOT, PV		AS, RPA, LPA	SVR, PVR	AD
4				SVR, PVR, AS	AD, VS
5				SVR, PVR, AD	
6	VS			SVR, PVR	AD
7		relationship of the great arteries		SVR, PVR, ventricular loop, AV and VA connection, RV, LV, RVOT, LVOT, PV, MPA, RPA, LPA, aortic valve, aortic root, aortic arch	AD
8				SVR, PVR, AS, PV	AD, VS
9				SVR, PVR, AS, PV	AD, VS
10				SVR, PVR, AD, ventricular loop, AV and VA connection, RV, LV, RVOT, LVOT, PV, MPA, RPA, LPA, aortic valve, aortic root, aortic arch	

*ND, “not-diagnostic”; SVR, systemic venous returns; PVR, pulmonary venous returns; AD, arterial duct; VS, ventricular septum; RVOT, right ventricular outflow tract; LVOT, left ventricular outflow tract; PV, pulmonary valve; AS, atrial septum; RPA, right pulmonary artery; LPA, left pulmonary artery; AV, atrio-ventricular; VA, ventricular-arterial; RV, right ventricle; LV, left ventricle; MPA, main pulmonary artery*.

Post-mortem micro-CT could not distinguish the integrity of ventricular septum from the presence of small or medium ventricular septal defect (case 1, 4, 6, 8, 9). On the other hand, it was able to define large ventricular septal defect (case 2, 3, 7, 10) and integrity of the ventricular septum (case 5). Post-mortem micro-CT could not define the ductus arteriosus in all except from 2 cases (case 5, 9).

Conventional autopsy could not define systemic and pulmonary venous returns (possibly due to specimen harvest), atrio-ventricular connection (case 7, 10), atrial septum (case 3, 4, 8, 9), ventricular septal defect (case 1, 4, 8, 9), right ventricular outflow tract and left ventricular outflow tract (case 7,10), pulmonary valve (case 7, 8, 9, 10), main pulmonary artery (case 3, 7, 10), left and right pulmonary arteries (case 3, 7, 10), aortic valve (case 7, 10), aortic root, and aortic arch (case 7, 10).

5 cases met the criteria for “challenging specimens” (case 4, 6, 7, 8, 10). In case 4, post-mortem micro-CT defined 23 out of 25 indices while conventional autopsy defined 20 out of 25 indices. In case 6, post-mortem micro-CT defined 23 out of 25 indices while conventional autopsy defined 22 out of 25 indices. In case 7, post-mortem micro-CT defined 23 out of 25 indices while conventional autopsy defined 8 out of 25 indices. In case 8, post-mortem micro-CT defined 23 out of 25 indices while conventional autopsy defined 19 out of 25 indices. In case 10, post-mortem micro-CT defined 24 out of 25 indices while conventional autopsy defined 8 out of 25 indices. 50/76 (65.7%) “not-diagnostic” indices belong to the group of the “challenging specimens.” For 42/50 (84%) indices, conventional autopsy was “not-diagnostic” whether micro-CT was diagnostic (“apparent advantage of micro-CT”). For 6/50 (12%) indices, nor micro-CT nor conventional autopsy were diagnostic (cases 4, 6, 7, 8: ventricular septum, arterial duct). For 2/50 (4%) indices, micro-CT was “not-diagnostic” whether conventional autopsy was diagnostic (“missense of micro-CT”; case 3, ventricular septum, relationship of the great arteries).

**Figure 1:**
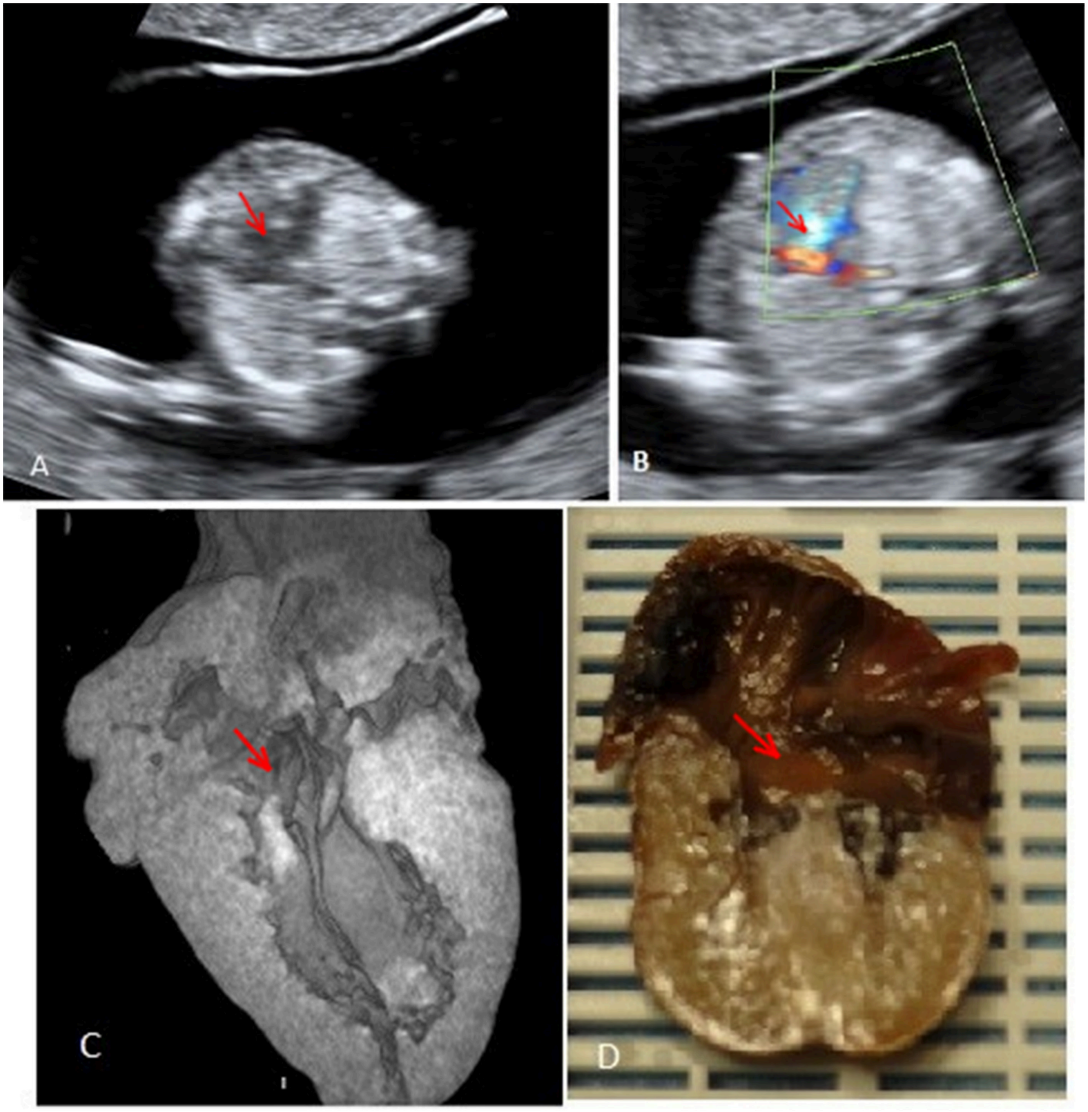
Case 1 (atrioventricular septal defect): **(A)** Prenatal fetal echocardiography: four chamber view showing the atrioventricular septal defect. **(B)** Prenatal fetal echocardiography: four chamber view with color Doppler confirmation of the defect. **(C)** Post-mortem micro-CT: four chamber view showing the atrioventricular septal defect. **(D)** Conventional autopsy: coronal section showing the four chamber view and the atrioventricular septal defect. Red arrows mark the atrioventricular septal defect.

**Figure 2:**
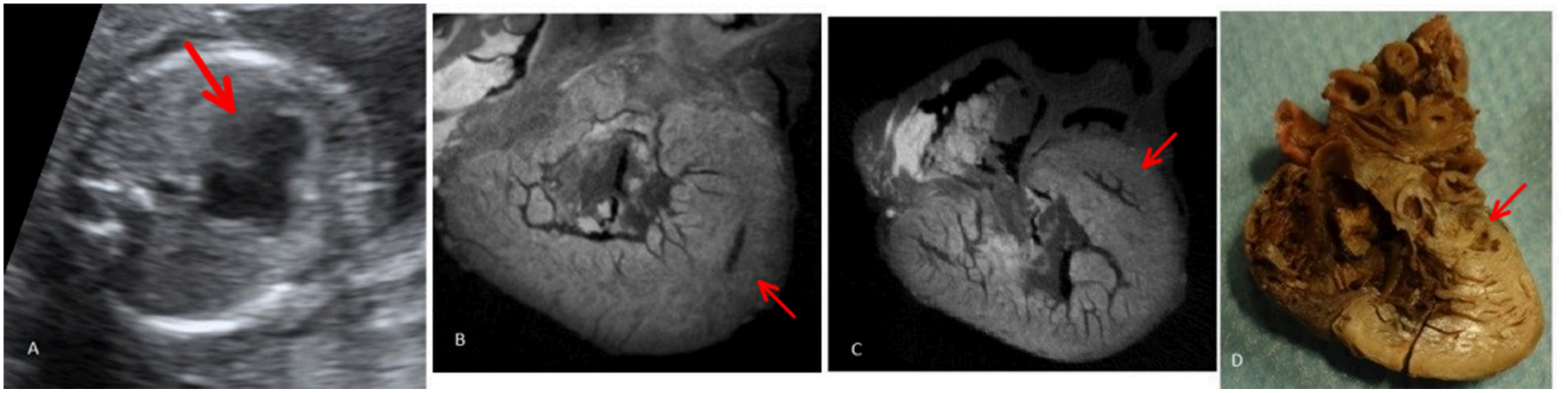
Case 2 (hypoplastic left heart syndrome): **(A)** Prenatal fetal echocardiography: four chamber view showing hypoplastic/atretic mitral valve and hypoplastic left ventricle. **(B)** Post-mortem micro-CT: short axis view at the level of the ventricle comparing right ventricle with hypoplastic left ventricle. **(C)** Post-mortem micro-CT: four chamber view showing the hypoplastic left ventricle. **(D)** Conventional autopsy: four chamber view showing hypoplastic left ventricle. Red arrows mark the hypoplastic left ventricle.

**Figure 3:**
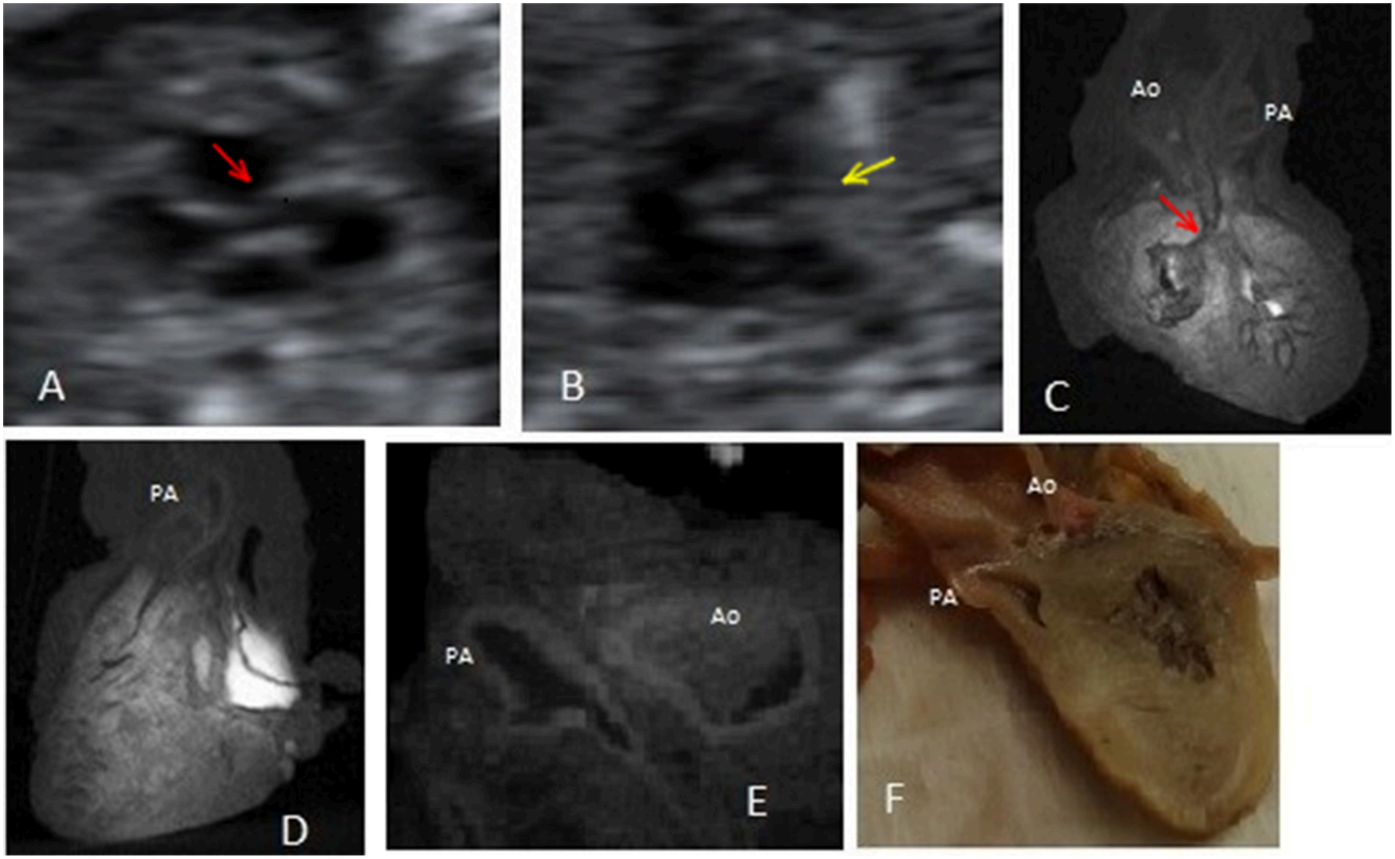
Case 3 (pulmonary atresia and ventricular septal defect): **(A)** Prenatal fetal echocardiography: conoventricular septal defect and an overriding vessel of unclear morphology. **(B)** Prenatal fetal echocardiography: short axis view at the level of the semilunar valve showing the absence of the pulmonary artery structure. **(C)** Post-mortem micro-CT: presence of a ventricular septal defect, a posterior overriding vessel and another anterior vessel. **(D)** Post-mortem micro-CT: the anterior vessel exits from the anterior, morphological right ventricle. **(E)** Post-mortem micro-CT: bifurcation of the anterior vessel. **(F)** Conventional autopsy: presence of two different outflow tracts. Red arrows mark the ventricular septal defect. Yellow arrow marks the absent pulmonary artery. Ao, aorta; PA, pulmonary artery.

Figures 1–3 give some examples of prenatal suspected congenital heart disease and post-mortem evaluation of samples.

## Discussion

To the best of our knowledge, this is the first study where human fetal hearts, with early and late prenatal diagnosis of congenital heart disease, undergo post-mortem evaluation by micro-CT and conventional autopsy by using segmental approach (10 cases, gestational age >13 weeks of gestation, prenatal diagnosis available for each case). Previous studies defined the feasibility of post-mortem micro-CT in evaluating murine (18) and human *ex vivo* pathological fetal heart (12, 13). Lombardi and colleagues demonstrated that post-mortem micro-CT provides similar information to conventional autopsy in case of normal heart and of prenatal suspected congenital heart disease by analyzing the four chamber view, the crux cordis, the atrioventricular valves and the three vessel cross sectional view of pulmonary artery, aorta, and systemic venous returns (6 cases, gestational age >15 weeks of gestation, prenatal diagnosis) (12). Hutchinson and colleagues showed that post-mortem micro-CT provide accurate definition of congenital heart disease by studying 21 indices of cardiac anatomy (5 cases, gestational age >17 weeks of gestation, no prenatal diagnosis) (13). Segmental approach is worldwide used to study the heart and to diagnose congenital heart disease. Therefore, we applied it to both post-mortem micro-CT and conventional autopsy and we compare the two techniques on its base.

The primary finding of this study is that post-mortem micro-CT, when compared to conventional autopsy, affords good agreement, sensitivity, and specificity in post-mortem evaluation of human fetal heart affected by congenital heart disease. Therefore, we can conclude that it has equal diagnostic power as compared to gold standard technique.

The secondary finding of this study is that post-mortem micro-CT defines morphological data even in challenging specimens, where autopsy lacks to. Indeed, for each case belonging to this subgroup of samples, post-mortem micro-CT was invariably able to define more indices as compared to conventional autopsy. Moreover, this subgroup of samples includes the majority (65.7%) of “not-diagnostic” indices, of which 84% are judged not evaluable by conventional autopsy but would be evaluable by micro-CT (“apparent advantage of micro-CT”).

Furthermore, post-mortem micro-CT is able to identify potentially prognostic important anatomic features (i.e., origin and position of the coronary arteries, anatomy of atrioventricular and semilunar valves) which are not dependably identified by conventional autopsy.

Technically speaking, post-mortem micro-CT does not jeopardize the diagnostic power of a subsequent conventional autopsy. After iodine preparation for micro-CT, we observed a slight reduction in weight of samples without modifications of tissue architecture and anatomic relation of intra-cardiac and extra-cardiac structure, as previously reported (12, 13). In addition, post-mortem micro-CT may guide pathologist in performing conventional autopsy, especially in small samples. In fact, images of post-mortem micro-CT allow the pathologist to decide where to cut and what to search for during the execution of challenging conventional autopsies. All this notwithstanding, conventional autopsy may not reach sufficient diagnostic power in all specimens.

Another possible advantage of micro-CT is represented by its non-destructive nature. It acquires a data set of images that can be reviewed and reconstructed many times and by different specialists, facilitating multidisciplinary discussion and comparison.

There are factors which lower diagnostic power of post-mortem micro-CT, as previously reported (18), including: poor quality of the specimens; post-mortem native tissue modification; and suboptimal preparation of samples. Poor tissue quality reduces the diagnostic accuracy of post-mortem micro-CT for small extra-cardiac structure (i.e., systemic and pulmonary venous returns). Post-mortem modifications of the muscular structure reduce the diagnostic accuracy of post-mortem micro-CT for small and medium ventricular septal defect. The definition of systemic and pulmonary venous returns and ventricular septal defect remains challenging also for conventional autopsy. Some of these indices (i.e., small ventricular septal defect) have no prognostic impact on postnatal care.

Limitations to the present study exist, including: the small population; the heterogeneity of samples, the absence of a control group (normal human fetal heart coming from early and late termination of pregnancy); the non-blinded comparison between techniques.

Nonetheless, the present pilot experience is the largest to date employing this innovative technique for post-mortem diagnosis of human congenital heart disease detected early in pregnancy.

In conclusion, we have preliminary evidence that post-mortem micro-CT gives at least equivalent information on structural heart defects as compared to conventional autopsy in *ex vivo* isolated human fetal hearts. In small specimens, where conventional autopsy has limited accuracy, post-mortem micro-CT provides more information on cardiac anatomy.

We conclude that micro-CT is at least equal to conventional autopsy for post-mortem confirmation of congenital heart disease and that it is superior to conventional autopsy in small specimens. More cases are needed to reach statistical power to confirm this finding and to define the threshold of weight and/or dimension under which conventional autopsy will be no more indicated. For further project, study population should consist in normal and pathological specimens and it should comprise enough samples from different gestational ages and enough samples of the same heart malformations. Double blinded study would be desirable for future investigations.

## Data Availability

The datasets generated for this study are available on request to the corresponding author.

## Ethics Statement

This study was carried out in accordance with the recommendations of local ethic committee (Comitato etico per la sperimentazione clinica delle province di Verona e Rovigo) with written informed consent from all subjects. All subjects gave written informed consent in accordance with the Declaration of Helsinki. The protocol was approved by the local ethic committee (Comitato etico per la sperimentazione clinica delle province di Verona e Rovigo).

## Author Contributions

CS contributed to conception and design of study, analysis and interpretation of data, drafting of manuscript. LR contributed to conception and design of study, drafting of manuscript, and final approval. VZ, RZ, FB, RS, CDP, and SH contributed to analysis and interpretation of data. FLR and GF contributed to final approval. CL and GBL contributed to conception and design of study, drafting of manuscript, funding, and gave final approval.

### Conflict of Interest Statement

The authors declare that the research was conducted in the absence of any commercial or financial relationships that could be construed as a potential conflict of interest.
